# The Evolution and Emergence of RNA Viruses

**DOI:** 10.3201/eid1605.100164

**Published:** 2010-05

**Authors:** Brian W.J. Mahy

**Affiliations:** Centers for Disease Control and Prevention, Atlanta, Georgia, USA

**Keywords:** RNA viruses, evolution, emergence, gravity model, spatial subdivision model, book review

This impressive monograph by Edward Holmes opens with a quotation from *La Peste,* by Albert Camus: “Everyone knows that pestilences have a way of recurring in the world; yet somehow we find it hard to believe in ones that crash down on our heads from a blue sky.” This apt quotation might lead the reader to believe that the evolution and emergence of RNA viruses in causing new diseases would be discussed, but in fact the book, as its title suggests, concentrates on how RNA viruses evolve and emerge at the molecular level, not how they cause disease.

In addition to explaining what is currently known about the origins of RNA viruses, the book describes the mechanisms of RNA virus evolution, RNA virus quasispecies, and comparative genomics, as well as interesting new concepts, such as phylogeography. This term refers to the spatial movement of a phylogenetic species, which can be described in various ways (Holmes lists 5), two of which are the gravity model and the strong spatial subdivision model. In the former, patterns of transmission are driven by major population centers before moving out to smaller populations (influenza virus). In the spatial subdivision model, no clear evidence of migration among populations is presented (hepatitis C virus), and genomic diversity is partitioned into a series of clades (types and subtypes).

Holmes argues persuasively that research in this area is limited by the size and detail of genome databases, combined with relevant epidemiologic and clinical information, such as precise geographic location, exact date of sampling, and transmission dynamics of the disease. The book is fully referenced and has a useful index, and I recommend it to those who have knowledge of and interest in the molecular biology of RNA viruses.

